# Advancements in Non-Thermal Processing Technologies for Enhancing Safety and Quality of Infant and Baby Food Products: A Review

**DOI:** 10.3390/foods13172659

**Published:** 2024-08-23

**Authors:** Nasim Pasdar, Parisa Mostashari, Ralf Greiner, Anissa Khelfa, Ali Rashidinejad, Hadi Eshpari, Jim M. Vale, Seyed Mohammad Taghi Gharibzahedi, Shahin Roohinejad

**Affiliations:** 1 Department of Agricultural Engineering and Technology, Payame Noor University (PNU), Tehran 19395-4697, Iran; nasiimpasdar@gmail.com; 2 Department of Food Science and Technology, Faculty of Pharmacy, Tehran Medical Sciences, Islamic Azad University, Tehran 19419-33111, Iran; mostashari.p.63@gmail.com; 3 Max Rubner-Institut, Federal Research Institute of Nutrition and Food, 76131 Karlsruhe, Germany; ralf.greiner@mri.bund.de; 4 École Supérieure de Chimie Organique et Minérale (ESCOM), Université de Technologie de Compiègne (UTC), EA 4297 TIMR, 1 Allée du Réseau Jean-Marie Buckmaster, 60200 Compiègne, France; a.khelfa@escom.fr; 5 Riddet Institute, Massey University, Private Bag 11 222, Palmerston North 4442, New Zealand; a.rashidinejad@massey.ac.nz; 6 Department of Food Science and Technology, Oregon State University, Corvallis, OR 97331, USA; eshparih@oregonstate.edu; 7 Department of Food, Nutrition and Packaging Sciences, Clemson University, Clemson, SC 29634, USA; jvale@zoomessence.com; 8 Institute of Materials Science, Faculty of Engineering, Kiel University, 24143 Kiel, Germany

**Keywords:** infant formulas, baby foods, food safety, quality control, functional foods, nutrition, probiotics, non-thermal processing

## Abstract

Breast milk is the main source of nutrition during early life, but both infant formulas (Ifs; up to 12 months) and baby foods (BFs; up to 3 years) are also important for providing essential nutrients. The infant food industry rigorously controls for potential physical, biological, and chemical hazards. Although thermal treatments are commonly used to ensure food safety in IFs and BFs, they can negatively affect sensory qualities, reduce thermosensitive nutrients, and lead to chemical contaminant formation. To address these challenges, non-thermal processing technologies such as high-pressure processing, pulsed electric fields, radio frequency, and ultrasound offer efficient pathogen destruction similar to traditional thermal methods, while reducing the production of key process-induced toxicants such as furan and 5-hydroxymethyl-2-furfural (HMF). These alternative thermal processes aim to overcome the drawbacks of traditional methods while retaining their advantages. This review paper highlights the growing global demand for healthy, sustainable foods, driving food manufacturers to adopt innovative and efficient processing techniques for both IFs and BFs. Based on various studies reviewed for this work, the application of these novel technologies appears to reduce thermal processing intensity, resulting in products with enhanced sensory properties, comparable shelf life, and improved visual appeal compared to conventionally processed products.

## 1. Introduction

The first days of life, from before birth to a child’s second birthday, are a pivotal period for human development [1,2]. Infancy is a period of rapid growth, second only to fetal life, and maintaining optimal nutrition is necessary during this time [3]. This critical period of growth is marked by numerous psychological, physical, and mental changes, including the development of digestive and immune functions, as well as the composition of the gut microbiota [4,5].

Nutrition during infancy plays a vital role in preventing non-communicable diseases [6,7]. Insufficient nutrient intake is a primary cause of postnatal growth restriction in neonates and the post-neonatal period [5]. Several studies have reported that undernutrition is responsible for 45% of all deaths among children under 5 years of age [8]. Breastfeeding is widely recognized as the nutritional gold standard for infants. The World Health Organization (WHO) recommends breastfeeding for at least six months following birth. Several studies have shown that breast milk protects against numerous diseases and enhances the intellectual development of children [8,9]. However, in situations where breastfeeding is unavailable, inappropriate, or inadequate, milk-based infant formulas (IFs) are used as a substitute for breast milk [1].

IFs, formulated to mimic mature human milk, are designed to provide infants with optimal nutrition for their development and growth [10]. Baby foods (BFs) are a type of soft, easily digestible food specially formulated for infants up to 12 months and young children from 1 to 3 years old. They are designed to provide essential nutrients required for growth and development during the critical period when infants transition from breast milk or formula to solid foods [11]. The global market value for BF and IF products exceeded USD 88 billion in 2022 and is expected to reach USD 150 billion by 2032, with a CAGR of over 5.3% [12]. However, despite the increasing demand for commercial IFs and BFs worldwide, concerns remain regarding their safety and efficacy. Commercial IFs and BFs may become contaminated with various microbes at different stages of processing. In many instances, IFs are intrinsically contaminated due to the addition of thermally sensitive micronutrients without prior heat treatment, which is necessary to meet regulatory standards. Consequently, these raw ingredients serve as potential pathways for bacterial transmission [8]. Furthermore, with regard to liquid infant products, the drying zone as well as the containers used for filling act as vectors for biological transmission.

Generally, heat sterilization ensures microbiological safety and product stability [13]. However, excessive heating can adversely affect the sensory, biophysical, and nutritional characteristics of infant products. The main irreversible changes that occur during conventional thermal treatment of macronutrients in infant food include protein denaturation and aggregation, lipid–protein interactions, sugar isomerization, and various chemical reactions [14]. The Maillard reaction is particularly critical, producing toxicants such as acrylamide, heterocyclic amines, and certain polycyclic aromatic hydrocarbons, which are associated with the degradation of nutritional values and ratios [15,16]. Balancing food safety with minimizing heat treatment losses remains a significant challenge for IF and BF manufacturers. The limitations of traditional food processing technologies have led the food industry to seek alternative processing methods.

**Figure 1:**
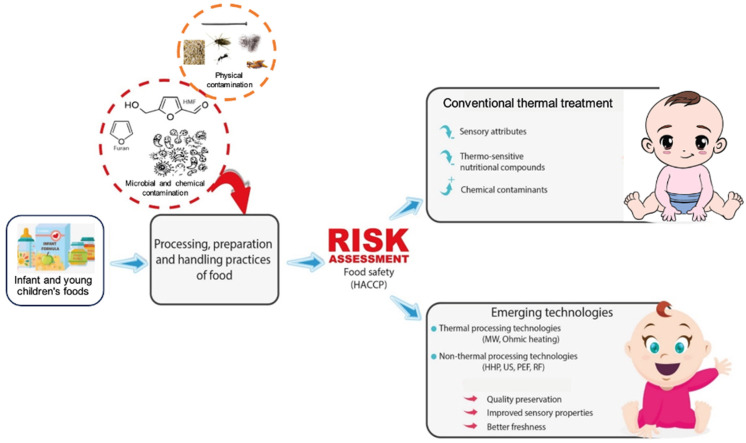
Comparison of conventional and emerging processing technologies in influencing sensory attributes, nutrient content, and freshness of infant and young child foods.

Non-thermal food processing technologies offer promising approaches to balancing microbiological and chemical safety, as well as sensory and nutritional properties in IF and BF production. Pioneering technologies such as high-pressure processing (HPP), ultrasound (US), pulsed electric field (PEF), and radio frequency (RF) are leading research in food processing for newborns and children. This review aims to assess the risk associated with critical chemical and microbial contaminants in food intended for infants and young children. It also highlights the application of non-thermal technologies as alternatives to conventional heat processing, ensuring the safety, quality, and nutritional content of infant and baby products at both pilot- and industrial-scale production levels (Figure 1).

## 2. Infant and Young Children’s Food Categories

**Figure 2:**
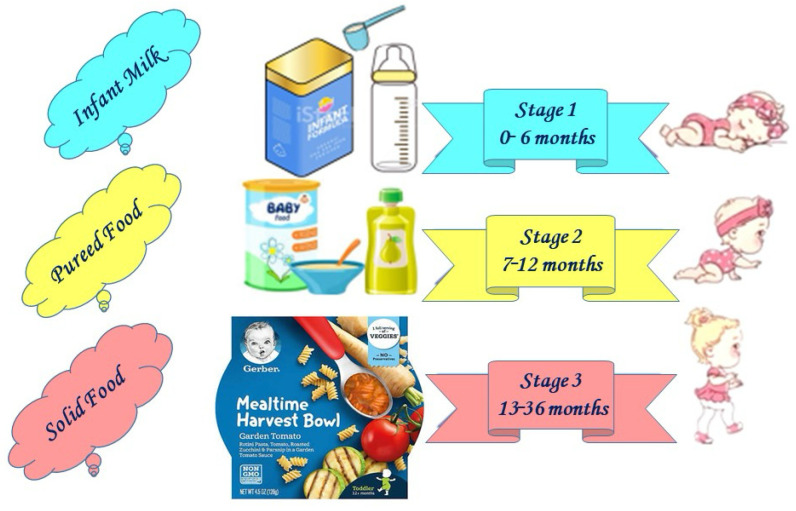
Examples of food products for different stages of infant and baby growth.

The consumer population of infants and young children under 36 months of age is typically divided into three stages: stage one (0–6 months, infants), stage two (7–12 months, follow-up infants), and stage three (13–36 months, young children) [17]. Infant products are specifically formulated to meet the distinct developmental stages of infants, each stage having unique nutritional requirements as defined by the recommended dietary allowance (RDA). Figure 2 illustrates various stages of IFs and BFs in accordance with child growth. The main foods consumed by infants and young children under 36 months of age include infant formula, complementary foods, and water are discussed as follows:

### 2.1. Infant Formula

The European Society of Pediatric Gastroenterology, Hepatology, and Nutrition (ESPGHAN) Committee on Nutrition, along with the Scientific Committee on Food of the European Commission, provides recommendations and regulations governing the nutrient composition of infant formula products. According to their guidelines, “infant formula” is designed as a suitable substitute for breastfeeding during the first 4–6 months (stage one). “Follow-on formula” (FOF) is intended for infants aged 6–12 months (stage two), while “toddler formula” or “follow-up formula” (FUF) is appropriate for children between 12 and 36 months (stage three) [18,19]. To meet the nutritional needs of infants aged 3 to 36 months as an alternative to breastfeeding, IFs must adhere to the nutritional requirements outlined by the WHO and the United Nations International Children’s Emergency Fund (UNICEF). 

Infant formulas are formulated using bovine milk, milk from other sources, or a combination thereof, with or without additional ingredients, to fulfill the nutritional needs of infants. These formulas are available in various formulations, including skimmed versions; those diluted with vegetable oils; fortified with vitamins, minerals, and iron; and those containing rice, carob, or soy for specific medical requirements [9,20]. Generally, IFs are available in three forms: powder, liquid concentrate, and ready-to-feed. While ready-to-feed bottles contain pre-diluted liquid formula, concentrated liquids and powder forms need to be mixed with water, which can include mineral water, bottled water, or tap water [21].

While a wide range of formula brands and types are available on the market (Table 1, [22,23,24,25,26,27,28,29,30,31,32,33,34,35,36,37,38,39,40,41]), there is no universally appropriate formula for all babies. The type of powdered milk recommended by pediatricians depends on a child’s nutritional needs or specific infant risks. Several leading manufacturers in the global infant nutrition market include Abbott (Chicago, IL, USA), Baby Gourmet (Calgary, AB, Canada), Danone (White Plains, NY, USA), Reckitt Benckiser Group PLC (Slough, UK), Nestlé (Vevey, Switzerland) [11], SMA^®^ Nutrition (Gatwick, UK), Friesl (Amersfoort, The Netherlands), Kraft Heinz (Sharpsburgh, PA, USA), HiPP GmbH & Co. (Pfaffenhofen an der Ilm, Germany), Vertrieb KG (Pfaffenhofen, Germany), and Arla Food (Viby, Denmark).

### 2.2. Complementary Foods

Complementary feeding, as defined by the WHO, is “a process starting when breast milk alone is no longer sufficient to meet the nutritional requirements of infants, and therefore other foods and liquids are needed, along with breast milk” [42]. This process, which includes continued breastfeeding, typically occurs between 6 to 23 months of age [43]. Commercial complementary food products are formulated to provide essential micronutrients and macronutrients needed during this stage. These products can be manufactured using simple techniques such as malting, popping, and fermentation, as well as modern food-processing technologies like roller drying, extrusion cooking, and non-thermal processing. Some of the most common commercially prepared IFs include iron-fortified cereals in varieties such as rice, oats, barley, wheat, mixed-grain, and grain with fruit. Additionally, there are juices, including those specifically for infants, citrus juice, canned juice, vegetable or fruit puree, and specially prepared meats for infant consumption [44].

## 3. Assessment of Hazards and Safety Measures in Infant and Baby Foods

The safety and quality of infant food are major concerns for parents and public health authorities [8]. Children are particularly vulnerable to foodborne illnesses, making the safety of food during processing, preparation, and handling critical to their health. There are three main types of hazards in foods that contribute to outbreaks: biological, physical, and chemical. These hazards are discussed in detail below:

### 3.1. Microbial Hazards

The nutrient content of infant and child foods makes them excellent growth media for bacteria. Therefore, any pathogen that remains after processing or contamination can rapidly multiply under optimal conditions [10]. During 2004–2006, the FAO/WHO consultation group identified the most common microorganisms associated with contamination in infant food. These include *Salmonella enteritidis*, *Cronobacter* sp., *Enterobacter agglomerans*, *Enterobacter cloacae*, *Klebsiella pneumoniae*, *Klebsiella oxytoca*, *Citrobacter freundii*, *Citrobacter koseri*, *Escherichia coli*, *Hafnia alvei*, *Acinetobacter* sp., *Serratia* sp., *Bacillus cereus*, *Clostridium botulinum*, *Clostridium perfringens*, *Clostridium difficile*, *Listeria monocytogenes*, and *Staphylococcus* sp. Among these, *Salmonella enterica* and *Cronobacter* sp. were identified as the most concerning pathogens, falling under hazard category A [45,46].

The resistance of microbial cells to heat and other processing technologies is influenced by various factors. These include the type of microorganism, with spores generally exhibiting greater heat resistance compared to vegetative cells. However, there can also be substantial variations in resistance among different species and strains of microorganisms. Additionally, the physiological condition of the cells and the conditions they are exposed to prior to treatment significantly impact the activation of resistance mechanisms, rendering bacteria more resilient to preservation and processing methods. In addition to microbial-specific factors, there are product-specific factors, often referred to as intrinsic factors. These intrinsic factors encompass characteristics such as pH, water activity, and compounds that can either hinder microbial growth or shield them from other stressors [47,48].

### 3.2. Physical Hazards

Physical hazards in IFs and BFs involve foreign objects that inadvertently enter products during various stages of production, posing significant risks like choking or physical injury to infants [49]. Such hazards can include metal fragments originating from broken machinery components, such as worn blades, loose screws, or damaged processing tools [50]. Glass shards may also be introduced through breakage in jars or bottles used during packaging. Plastic particles can result from damaged packaging materials or worn-out equipment parts, while stones, seeds, or hard particles can remain if raw ingredients like fruits, vegetables, or grains are not adequately cleaned or processed [51]. In meat-based BFs, small bone fragments can be a concern if deboning processes are not thorough. Wood splinters from storage pallets or crates and rubber pieces from deteriorating seals, gaskets, or other machine parts can also find their way into the food. These physical hazards are dangerous for infants, given their underdeveloped chewing ability and smaller airways [52]. To mitigate these risks, comprehensive quality control systems must be in place, including the use of metal detectors, X-ray scanners, fine sieves, and routine equipment maintenance [52]. Additionally, robust raw material inspection protocols and effective cleaning processes are essential for minimizing the introduction of such physical contaminants, ensuring that IFs and BFs remain safe and free from potentially harmful foreign objects [50,51,52].

### 3.3. Chemical Hazards

Foods and formulas designed for infants may contain toxic substances originating from raw materials, processing, or the formation of neo-formed contaminants. The high temperatures necessary for microbial inactivation can result in nutrient loss and the formation of undesirable compounds. Thermal processing of food products can produce toxic substances known as food-processing contaminants [53,54]. The compounds include furan and its methyl derivatives, 5-hydroxymethylfurfural (HMF), acrylamide, 3-monochloropropanediols (3-MCPD) esters, glycidyl esters, etc. [55,56]. The carcinogenic and genotoxic effects of these chemicals have been demonstrated in animal studies. Given these findings, the health risks linked to dietary exposure to food-processing contaminants are a significant concern in infants and toddlers due to their heightened sensitivity [57]. Due to higher rates of gastrointestinal uptake, an incomplete blood–brain barrier, an undeveloped detoxification system, and a large consumption of food relative to body mass, infants are more susceptible to food contaminants than adults [23,58]. The most common and problematic chemical contaminants found in commercial IFs and BFs are given as follows:

#### 3.3.1. Furan

Furan (C_4_H_4_O) is a lipophilic contaminant primarily formed during the thermal processing of foods at 150 to 200 °C [59]. This compound is heterocyclic, volatile, and has a low molecular weight, with a boiling point close to room temperature (31.36 °C) [60]. A recent report by the International Agency for Research on Cancer and the U.S. National Toxicology Program classified furan as a “possible carcinogen of humans” (group 2B) [61]. Several pathways contribute to furan formation, including Maillard reactions, thermal dissociation of amino acids, oxidation of ascorbic acid, and oxidation of PUFAs [62]. One of the key reactions is the Maillard reaction, which involves the reduction of amino acids and sugars, producing volatile compounds associated with processed foods [53].

Historical data from the Food and Drug Administration (FDA) and European Food Safety Authority (EFSA) have revealed that furan formation can be detected in a wide range of commercially available infant and child foods that have undergone thermal processing. This includes IFs, ready-to-eat products, canned foods, and jarred foods [63]. The primary sources of dietary furan exposure are jarred food for infants [64], and breakfast cereals for children [65]. Furan concentrations can vary depending on the food composition. The furan content of IFs, infant foods, and BFs ranges from 2.5 to 27 μg/kg (mean: 12 μg/kg), 1.3 to 87.3 μg/kg (mean: 27 μg/kg), and 1 to 112 μg/kg (mean: 28 μg/kg), respectively [66]. According to Jestoi et al. [67], the average furan content in BFs based on their ingredients was 9.2, 37.0, and 49.6 mg per kilogram for products containing fruit, vegetables, and meat, respectively. The presence of furan in BFs continues to raise serious food safety concerns for regulatory authorities. Furan production is primarily limited to commercially sterilized foods, while freshly cooked, home-prepared foods are free from furan [68]. 

#### 3.3.2. 5-Hydroxymethylfurfural (HMF)

HMF (C_6_H_6_O_3_) is one of the most prominent furan derivative compounds. This cyclic aldehyde is commonly formed as an intermediate in the Maillard reaction. It is primarily synthesized through the thermal degradation of hexoses in the presence of proteins or amino acids. Additionally, it can be obtained by acid-catalyzing the thermal dehydration of various C_6_-based carbohydrates, such as glucose and fructose [69]. HMF serves as an indicator of quality deterioration resulting from excessive heat treatment or improper storage conditions [70]. It can be produced as a byproduct of side chemical reactions during the temperature sterilization and browning of dairy infant products. Given that these precursors are the sole source of lysine for infants, the Maillard reaction is considered unacceptable in infant milk [71].

According to a study investigating the Maillard reaction in commercial IF_S_ using HMF as a thermal indicator, the amount of HMF was found to be in the range of 62–510 μg per 100 g [72]. Another study examined the potential levels of HMF in various IFs and BFs available on the market, including milk-based infant formulas, follow-on milk, and cereals [73]. The study found that IFs contained a higher mean level of HMF compared to follow-on milk. Fruit-based BFs tend to have higher levels of HMF due to the accelerated breakdown of carbohydrates facilitated by lower pH levels. For instance, a study conducted by Vella et al. [23] reported HMF concentrations in IFs ranging from 0.89 mg/kg to 144 mg/kg, with prune-based IFs displaying the highest concentration. In another study, furan derivatives were detected in both homemade and commercially produced samples of BFs, including those containing vegetables, fruits, and meats [70]. This analysis demonstrated significant variability in the average levels of furan derivatives across samples, with plum-based BFs exhibiting the highest HMF concentration at 343.10 μg per gram.

#### 3.3.3. Acrylamide, 3-MCPD Esters, and Glycidyl Esters

The formation of acrylamide, 3-MCPD esters, and glycidyl esters in BFs and IFs is primarily influenced by high-temperature processing. Acrylamide is produced via the Maillard reaction, mainly in carbohydrate-rich foods (e.g., fried potato, bread) at temperatures above 120 °C, where reducing sugars and the amino acid asparagine react. In IFs, acrylamide can form during spray drying at temperatures more than 150 °C, especially when the product has low moisture content and a slightly alkaline pH [74]. Acrylamide levels in infant snack foods (e.g., teething biscuits, puffs, crackers, and rusks) were examined using an exposure assessment in the United States from 2011 to 2015. Researchers found that infant snack foods significantly contribute to acrylamide intake compared to infant jarred foods. The acrylamide content in these foods ranged from 5 to 1788 µg/kg, with an average concentration of 165.1 µg/kg. Among these, the maximum average acrylamide levels were assessed in infant crackers (826.9 µg/kg) and teething biscuits (414.5 µg/kg) [75]. However, the EFSA panel on “Contaminants in the Food Chain” reported mean acrylamide levels in European countries as 111 µg/kg for baby biscuits–rusks, 231 µg/kg for crackers and biscuits, and 201 µg/kg for wafers [76]. 3-MCPD esters and glycidyl esters are typically formed in fat-containing products during the oil refining process. Specifically, these contaminants arise during the deodorization stage, which involves temperatures above 200 °C. The presence of chlorine-containing compounds in the oil, combined with prolonged heating, escalates the development of 3-MCPD esters. Glycidyl esters are similarly formed at these elevated temperatures, mostly when the product is exposed to conditions that favor the breakdown of triglycerides, leading to free fatty acids and glycerol that react to form these harmful compounds [77,78]. Spungen et al. [77] analyzed a small convenience sample of IFs with the US Food and Drug Administration to determine levels of 3-MCPD esters and glycidyl esters. The findings were used to estimate exposure levels in infants aged 0–6 months consuming these IFs. Based on average concentrations across all formulas, the estimated exposures were 7–10 µg/kg body weight per day for 3-MCPDE and 2 µg/kg for glycidyl esters. After analyzing Ifs produced by specific manufacturers, average exposure estimates varied between 1 and 14 µg/kg per day for 3-MCPDE and 1 and 3 µg/kg per day for glycidyl esters [77]. In BFs like fruit purees, these substances can be produced during extended heat treatments or in processes, involving direct contact with heated surfaces, such as in retort sterilization or drying steps, where fat content and processing duration are key factors [79].

## 4. Conventional Thermal Processing

The use of thermal processing has been popular for over 150 years as a means of food preservation [80]. This technique is still widely used for microbial decontamination of food products to destroy pathogenic microorganisms, preserve the product, extend shelf life, and ensure consumer safety [81]. Thermal processing technologies can be categorized as direct or indirect, each with different heat transfer mechanisms. In the indirect system, the product is separated from the heating fluid (warm water or steam under pressure) by a wall, whereas in the direct system, the product is mixed with the heating fluid (steam) [82]. Compared to indirect heating, direct heating entails a lower thermal load on the product, heating and cooling more rapidly and causing fewer thermally induced changes throughout the product. However, direct systems are challenged by the requirement for culinary-grade steam, lower heat regeneration capacity, and product dilution concerns [83]. 

The most employed methods for sterilizing powdered and liquid infant food products include indirect high-temperature short-time and direct ultrahigh temperature (UHT) techniques. Sterilization processes can occur after the product is packaged in cans or glass bottles, or through direct or indirect heating before the product is packaged in sterile glass, metal cans, plastic, or cardboard containers through aseptic filling [84,85]. Several studies have reported the sterilization of IFs and BFs (e.g., fiber-rich vegetables, fruit puree) using conventional thermal methods such as hot water baths (85–95 °C, 5–30 min) [86]; shell, plate, or tube heat exchangers (115–140 °C, 3–15 s) [87,88]; and retort/autoclaves (121–130 °C, 10–40 min, depending on the product and packaging) [57,60].

Conventional treatments with high temperatures are widely recognized as effective methods for ensuring the destruction of microorganisms in infant and young child food. However, their application can have negative thermal effects on food quality, leading to the loss of heat-sensitive nutrients and adverse changes in sensory attributes. In a study by Sun et al. [89], various thermal processes, including pasteurization (72 °C, 15 s), ultra-pasteurization (95 °C, 30 s), UHT instantaneous sterilization (130 °C, 5 s), and in-container sterilization (120 °C, 15 min), were used to investigate the structural and physicochemical properties of model IFs. The results showed that in-container sterilization processing induced the most pronounced Maillard reaction, as indicated by changes in color and thermal properties. Among the tested samples, in-container sterilization had the most detrimental impact, followed by ultra-pasteurization, UHT, and pasteurization, in that order. Given these adverse effects, there is an urgent need to develop effective treatments and procedures to ensure the microbiological safety of IFs and BFs while preserving their nutritional and sensory quality. Globally, research on innovative non-thermal processing technologies has garnered significant attention as a promising alternative to conventional thermal processing methods.

## 5. Non-Thermal Processing Technologies

Innovative non-thermal approaches such as HPP, PEF, RF, and US are recognized for their effectiveness in ensuring the safety of IFs and BFs by eliminating pathogenic microorganisms (Table 2, [22,90,91,92,93,94,95,96,97,98,99,100,101,102,103,104,105,106,107,108,109,110,111,112,113]). A key goal of these non-thermal technologies is to maintain product quality by minimizing the thermal impact on the manufacturing process. This approach results in improved sensory attributes and comparable shelf life, leading to a more appealing visual appearance and a better taste experience [114]. 

### 5.1. High-Pressure Processing (HPP) Technology

HPP is a food preservation method that uses elevated pressure to deactivate microorganisms and enzymes, thereby extending the shelf life of food products [115,116]. HHP technology is based on two fundamental principles: Le Chatelier’s principle and the isostatic principle. Le Chatelier’s theory states that pressure-induced volume reduction alters structural characteristics, while the isostatic principle maintains that pressure is uniformly distributed and proportional in all fluid foods [117,118]. Industrially, this method is predominantly used for processing liquids and solids but is unsuitable for dried milk powder or cereals due to their low moisture content [119].

An HHP technology system involves injecting water into a high-performance cylinder, where foods to be treated are pre-packaged in a flexible material that transmits the water pressure. Solid food products are vacuum-sealed, while liquid food products use a headspace-free seal [116]. HHP processing technology allows for simultaneous control of three processing parameters—pressure, temperature, and time—offering significant process design flexibility [120,121]. This typically entails subjecting target food products to pressures of 400–600 MPa at temperature ranges of 45 °C or refrigeration, with holding times of 1.5–6 min [48,122].

Unlike heat treatment, pressure treatment is not influenced by product size or geometry, leading to reduced processing times. Previous reports indicate that one of the key advantages of HHP for the IFs/BBF industry is a decrease in unwanted food processing contaminants and microbial populations [115,123]. A major mechanism for microbial inactivation involves the disruption of non-covalent bonds and damage to cytoplasmic ribosomes and cell membranes. HHP can induce several phenomena simultaneously (e.g., disruption of cell walls and membranes, chemical reactions, enzyme activation or inactivation, and protein modification, such as denaturation and gel formation), thereby influencing the overall microbial load [124,125].

High-pressure pasteurization effectively eliminates spoilage bacteria, yeasts, and molds but is ineffective against spores [25]. To enhance the sterilization and pasteurization processes, high pressures (600 MPa) combined with high temperatures (90–121 °C) are used to inactivate spores, a method known as “high-pressure thermal sterilization” (HPTS) [126,127]. The combination of high pressure and high temperature produces a synergistic effect that reduces processing time, minimizes undesired food processing contaminants, improves food quality, and eliminates microorganisms [128]. In 2015, the FDA approved pressure-enhanced sterilization, allowing for sterilization temperatures below 121.1 °C at 600 MPa [129]. 

Numerous studies have demonstrated the effectiveness of HPP, either alone or in combination with other technologies, for processing various BFs. For instance, Kultur et al. [90] used HHP for the pasteurization of fruit purees for babies, achieving pathogen inactivation without generating chemical contaminants such as furan. HPP treatments were conducted at 25, 35, and 45 °C, and pressures of 200, 300, and 400 MPa, for treatment durations of 5, 10, and 15 min. Significant reductions (around 6 log_10_) in mesophilic aerophiles and yeasts/molds were achieved at 400 MPa and 45 °C for 15 min without furan formation. Kultur et al. [91] also investigated the potential of HPP for inactivating total mesophilic aerobic bacteria, total yeasts and molds, and reducing furan formation in vegetable-based IFs. They highlighted the synergistic effects of processing parameters such as time, temperature, and pressure in microbial inactivation. The effectiveness of microbial reduction depended on the specific microorganism and the HPP conditions. For example, treatment at 400 MPa and 45 °C for 15 min led to complete inactivation of all microorganisms, with no furan detected in any sample. The use of low temperatures in processing fruit and vegetable baby products helps preserve their sensory characteristics.

Li et al. [92] evaluated the nutritional and functional properties of soy protein isolate for IFs using HHP treatment. They found that various functional attributes, such as solubility, water holding capacity, foaming capacity, and emulsification activity index, were enhanced at lower pressure and time levels but diminished at higher levels. Foaming stability decreased with increasing pressure and time, while the emulsification stability index decreased with higher pressure. Soy protein isolate gels exhibited improvements in springiness, hardness, and adhesive force with longer treatment times and increased pressure, although these improvements were less pronounced than those observed in the control group. Furthermore, HHP induced conformational changes in the tertiary and/or quaternary structure of soy protein isolate [93].

Bu and Li [130] reported that the HHP-treated sample exhibited a higher swallowing response and greater in vitro digestibility compared to the control. Gel electrophoresis indicated that glycinin was more stable under pressure than β-conglycinin, and high molecular weight subunits were formed through disulfide interactions at higher treatment levels. In another study, Cetin-Karaca et al. [131] combined high pressure with *trans*-cinnamaldehyde to assess the effectiveness of HPP in deactivating *B. cereus* spores in reconstituted powdered infant formula milk (RPIFM) under optimal conditions (5 min at 600 MPa). The products were then stored at 23 °C and 7 °C for 4 and 6 weeks, respectively. The combined method demonstrated significant antimicrobial activity by eliminating vegetative cells and *B. cereus* spores in infant foods stored at room temperature and under refrigeration. HPP at 600 MPa reduced pathogen populations by two logs and combining HPP with 0.1% *trans*-cinnamaldehyde resulted in a three-log reduction. Long-term storage of RPIFM at room temperature led to decreased pH and increased microbial growth. The combination of HPP and *trans*-cinnamaldehyde was considered a robust antimicrobial alternative to thermal treatments and artificial preservatives. Cetin-Karaca et al. [94] found that a combination of HPP at 600 MPa for 5 min, 0.05% *trans*-cinnamaldehyde, and 1% chitosan, with storage temperatures of 7, 23, and 45 °C, led to the complete elimination of *Cronobacter sakazakii* after 4, 6, and 2 weeks, respectively. In all HPP treatments, the colony-forming units per milliliter (CFU/mL) decreased by at least 5.5 logs CFU/mL, compared to maximum reductions of 2.1, 1.1, and 3.7 logs CFU/mL without HPP treatments at 7, 23, and 45 °C. Moreover, sensory testing did not reveal a significant difference between the treatment group and the control group.

It has been documented that thermal processing and high-pressure thermal processing (HPTP) of pear purees, with or without citric acid, can inhibit oxidative enzymes such as peroxidase (POD) and polyphenol oxidase (PPO), while also improving their sensory properties [95]. Following HPTP treatment (600 MPa, 90 °C, 5 min) and thermal processing treatment (90 °C, 7 min) in acidified puree, POD was completely deactivated, whereas PPO was deactivated to a maximum of 60%. Therefore, pear puree treated with HP and HPTP was considered a suitable candidate for use in baby formulas due to its low pH, high antioxidant capacity, and reduced activity of oxidative enzymes.

Sevenich et al. [97] investigated the potential benefits of HPTS in the food industry, emphasizing its capacity to enhance food quality, diminish thermal impact, and lower the presence of undesired food processing contaminants such as furan. The study conducted laboratory-scale trials on specific food items to establish temperature–time combinations for achieving a 12 log_10_ inactivation of *Bacillus amyloliquefaciens*. These combinations were then applied in a scale-up process using a 55 L vessel equipped with a high-pressure, high-temperature system. The results demonstrated a significant decrease in furan levels, ranging from 41 to 98% compared to conventional retorting methods. Pilot-scale experiments confirmed these findings, with only one food product exhibiting instability after treatment. Additionally, storage trials (standardized method NF V 08-408) revealed that only two selected treatment conditions (107.5 °C, 9.8 min and 115 °C, 0.45 min at 600 MPa) resulted in an unstable product, specifically in the case of baby food puree. In conclusion, the study suggested that HPTS holds promise as a viable option for adoption within the food industry.

Gratz et al. [98] evaluated the effects of pressure-enhanced sterilization (PES) and ohmic (OH) technologies as alternatives to thermal retorting. Their goal was to enhance the quality of carrot puree for infant consumption by identifying optimal food safety process parameters. Both methods were found to reduce the thermal load on the product without compromising food safety or quality. This technology was observed to heat puree samples more rapidly and uniformly compared to conventional retorts, leading to lower C values. Additionally, PES treatments, besides their synergistic inactivation effect of temperature and high pressure, resulted in lower C values by lowering the processing temperature. As a result, color, bioactive compounds like carotenoids, and texture were better preserved, while food processing contaminants, particularly furan and its derivatives, were reduced.

Wang et al. [99] investigated the application of pressure-assisted thermal sterilization (PATS) to sterilize BFs, which offer higher nutritional value compared to products processed using conventional thermal methods. Their study focused on sterilizing BFs inoculated with *B. subtilis* spores using the PATS technique. The results indicated that the combination of temperature and thermal expansion pressure had a synergistic effect on microbial elimination, leading to significant reductions in *B. subtilis* levels and improved retention of ascorbic acid.

### 5.2. Radio Frequency (RF) Technology

Radio Frequency (RF) technology utilizes electromagnetic waves in the frequency range of 1 to 300 MHz. In RF heating, electromagnetic waves penetrate the food product, causing polar molecules (such as water) to oscillate and generate heat through molecular friction. The rapid and volumetric heating ensures uniform temperature distribution, which is particularly beneficial for foods with high moisture content. Key operational parameters include frequency (typically 13.56 or 27.12 MHz for food applications), power (adjustable depending on the food’s dielectric properties), and treatment time (from seconds to minutes). RF heating is primarily used for pasteurization, sterilization, drying, and thawing, providing rapid and uniform heating with minimal quality degradation [105,132]. In RF heating units (RF-H), electrodes do not directly contact the food to prevent Joule heating (OH heating). This technology is suitable for both solids and liquids due to its deep penetration capacity and rapid heating speed [133]. Numerous studies have demonstrated the successful use of RF heating for pasteurizing/sterilizing liquid or semi-liquid IFs. A recent experimental study by Lin et al. [103] confirmed the effectiveness of RF-enhanced traditional thermal processing (RF-assisted TTP) for pasteurizing PIFM. In this study, RF energy was applied to a tray containing PIFM, resulting in a cold spot observed in the center of the top layer. The RF treatment was used after inoculating the sample with *C. sakazakii* to heat it until the cold spot reached 65 °C at 27.12 MHz and 6 kW. Subsequently, the samples were held at 65 °C in a hot air oven for varying times. Following RF-assisted TTP at 65 °C for 21 h, *C. sakazakii* was reduced by approximately 5 logs. Qualitative analysis revealed no significant differences in solubility, wettability, digestibility, and color parameters between RF-assisted TTP and TTP. However, both treatments led to significant changes in moisture content, water activity, TBARS, and peroxide value.

Another study aimed to develop radio frequency dielectric heating (RFDH) processes to eliminate *Salmonella* spp. and *C. sakazakii* in contaminated nonfat dry milk (NDM) intended for infants [104]. They used a thermal death time (TDT) disk process to estimate the D-values (time required for a one-log reduction) of *Salmonella* spp. and *C. sakazakii* in NDM (low-heat: LH; high-heat: HH) at temperatures of 75, 80, 85, or 90 °C, and calculated the z-values (temperature increase required for a tenfold reduction in D-value). For *C. sakazakii*, D-values ranged from 5.37 to 24.86 min at different temperatures, while for *Salmonella* spp., D-values ranged from 4.55 to 24.94 min. The study found that both pathogens were inactivated similarly regardless of the treatment method (RFDH vs. conventional). This suggests that RFDH treatment could be used to achieve target temperatures for post-treatment lethality in NDM before packaging, in a high-speed and uniform manner, thereby reducing the risk of food safety issues.

Zhang, Zhu et al. [105] compared the effects of combined radio frequency and hot air treatment (RF-HA) with hot water treatment (HW) on the quality of PIFM for inactivating *C. sakazakii* by 5 log. Both heat treatment methods showed no significant differences in solubility or crude protein content. However, the increase in moisture content to 2.70 g/100 g (aw: 0.4) resulted in a significant decrease in the glass transition temperature of amorphous lactose, compromising the quality of PIFM. Lowering the moisture content in PIFM subjected to RF-HA treatment increased protein denaturation temperatures, leading to more stable protein structures. Additionally, RF-HA treatment induced less non-enzymatic browning compared to HW-treated samples. This was further supported by FTIR spectra, which indicated a lower rate of the Maillard reaction in samples treated with RF-HA. Moreover, RF had a significant effect on particle agglomeration in PIFM compared to conventional HW treatment.

In a study by Zhang, Xie et al. [106] a thermostatic RF system was used to deactivate *C. sakazakii* in PIFM. A proportional–integral–derivative controller was employed to maintain constant material temperature during holding. Similar to previous studies, dielectric material assistance and hot air were utilized to enhance RF heating uniformity. Results showed that the thermal resistance of *C. sakazakii* decreased with increasing water activity (0.2–0.4 at 25 °C) and temperature (55–70 °C). Combining RF with hot air pasteurization improved microbial inactivation compared to RF or material assistance alone, attributed to better temperature uniformity. Transmission electron microscopy (TEM) analyses and flow cytometry confirmed that RF treatment did not significantly affect the cell wall. RF processing has been shown to achieve significantly higher heating rates than traditional methods, making it a promising technique for pasteurizing PIFM efficiently. Another study by Wang et al. [107] evaluated the thermal death kinetics of *C. sakazakii* in PIFM using RF and hot water treatment. The research demonstrated that RF technology effectively disables *C. sakazakii* in packaged powdered IFs while maintaining product quality.

Lin et al. [108] studied the dielectric properties of packaging materials for PIFM to improve in-package pasteurization using RF and microwave heating (MWH). They investigated how temperature (20–80 °C), frequency (10 MHz–3 GHz), and main components (moisture, whey protein, fat, and lactose) influenced these properties. Results indicated that the loss factor and dielectric constant decreased with higher frequency, while the dielectric loss factor decreased with increased density, temperature, and fat content. The dielectric constant increased with density and main components. Lactose and whey protein exhibited positive dielectric properties due to ionic conduction, while fat had negative dielectric properties due to weak polar attraction. In another study, Zhong et al. [109] evaluated the impact of radio frequency heating (RFH; 90 °C, 5 and 10 min) on the microstructure, composition (fat distribution, protein oxidation), rehydration characteristics, and flow properties of PIFM. RFH treatment increased protein dityrosine concentration, free fat on powder surfaces, and powder porosity. Additionally, RFH improved flow ability and compressibility compared to the raw sample, although longer durations decreased rehydration ability, indicating lower solubility and smaller contact angles. The Guggenheim–Anderson–de Boer (GAB) model characterized water vapor sorption isotherms, showing that prolonged RFH duration increased C values (63% at 10 min).

Recently, Lin et al. [103] investigated the dielectric properties of PIFM, focusing on dipole loss (fat, lactose, and whey protein) and ionic loss at temperatures and frequencies ranging from 20–80 °C and 10 to 3000 MHz, respectively. They observed that the dielectric loss factor of PIFM increased with higher lactose and whey protein content but decreased with higher fat content. The ionic loss in PIFM increased with temperature but remained constant with frequency. Moreover, lactose, fat, and whey protein dipole loss followed the Debye equation, showing an increase with frequency up to approximately 1, 1, and 1.2 GHz, respectively, before declining. Increasing the whey protein content in reconstituted PIFM led to higher heating rates in RF fields and reduced lipid oxidation in processed PIFM.

PIFM can become sticky under unfavorable processing conditions, leading to negative impacts on its physicochemical and functional properties [134]. Zhang et al. [111] investigated the effect of RF dry heat treatment on PIFM stickiness. Spray-dried PIFM with a water activity of 0.28 was treated at 70 °C for 0–5 log inactivation of *C. sakazakii* for varying durations. The RF treatments significantly reduced water activity compared to untreated samples, with minimal changes in surface-free fat content (0.005–0.006 g/g powder) and lactose crystallinity (2–3%). The particle size of PIFM increased significantly initially and then stabilized after 23.3 min, indicating particle sticking occurred during the first pasteurization step. While a visible adhesion and flow of hot surface fat were observed during the 23.3 min pasteurization, the fat coverage decreased with longer treatment times. Additionally, the increased surface lactose coverage in treated PIFM reduced their water activity, affecting the glass transition. The study concluded that the enhancement in particle size after RF processing was due to free fat bridges on the particle surface, suggesting that RF technology can improve particle quality post-processing.

### 5.3. Ultrasound (US) Technology 

Ultrasound technology (US), a non-invasive and non-destructive technique, has gained significant attention in the food industry for its diverse applications. US processing employs high-frequency sound waves, typically above 20 kHz, to develop cavitation in liquid foods. This process generates localized high temperatures and pressures that disrupt microbial cell walls and enhance mass transfer. Key operational parameters include frequency (20 kHz–1 MHz), intensity (W/L), and duration (ranging from seconds to minutes). Lower frequencies are generally used for microbial control, while higher frequencies are applied for homogenization and emulsification [135,136]. This technology can modify the chemical, physical, and functional properties of food, thereby impacting its overall quality [137]. This phenomenon is attributed to cavitation in liquids, pressure changes in gases, and movement of liquids in solids [138]. In food processing, the US finds utility in process control, defect detection, property analysis, extraction efficiency improvement, drying, filtration, preservation, and meat tenderization [136,137].

Several studies have investigated the use of US technology in IFs and BFs. For example, Adekunte et al. [112] studied the quantitative impact of US as an alternative heating method for monitoring the inactivation kinetics of *C. sakazakii* in RPIFM. The optimization involved varying the amplitude (24.4, 30.5, 42.7, 54.9, and 61 m) and temperature (25 °C, 35 °C, and 50 °C), and the kinetics were analyzed using a modified Bigelow-type model. The combined use of US and temperature led to a significant reduction in the population of *C. sakazakii*. In another study, the effectiveness of US and conventional pasteurization methods in inactivating enzymes and microbes in pear juice for use in BFs was compared [22]. US was most effective at 25, 45, and 65 °C with 750 W power, a 20 kHz frequency, and 70% amplitude for 10 min. In contrast, conventional pasteurization required 95 °C for 2 min and 65 °C for 10 min. US pasteurization resulted in greater inactivation of microbes (yeast, mold, and total plate count) and enzymes at a lower temperature (65 °C for 10 min). Additionally, compared to conventional methods (95 °C for 2 min), the use of US retained more phenolic compounds.

Using sonication has been reported to tenderize meat by reducing myofibrillar proteins in muscle tissue and improving cohesion and water-holding capacity [139]. In a study by Luo et al. [113], US was investigated as a pretreatment method for raw meat to prepare infant meat puree at different power levels (200 W, 400 W, and 600 W, and 20 kHz) and durations (15, 30, and 45 min). Compared to the control, using US power at 400 W and 600 W resulted in decreased viscosity and hardness, and improved texture (firmer texture and higher water content) of the meat puree. Moreover, no significant difference in the digestibility of the meat puree in the gastric phase was observed, while the digestibility in the intestinal phase increased (80.85%) using US (600 W for 15 min).

### 5.4. Pulsed Electric Field (PEF) Technology

Pulsed electric field (PEF) is a non-thermal food preservation technology that involves applying short, high-voltage pulses to food products placed between two electrodes. These pulses induce an electric field that disrupts cell membranes through electroporation, leading to microbial inactivation. Operational parameters include voltage (typically 1 to 100 kV/cm), pulse duration (from microseconds to milliseconds), and the number of pulses (from hundreds to thousands). These parameters can be adjusted to optimize microbial inactivation while preserving food quality and nutrients [140]. Reversible permeabilization refers to the temporary opening of cell membranes, allowing for the extraction of intracellular compounds such as pigments, flavors, and nutrients. This process is often used in the food industry to improve the extraction efficiency of valuable components from plant and microbial cells [141]. Irreversible permeabilization, on the other hand, involves the permanent disruption of cell membranes, leading to cell death. This aspect of PEF is utilized for microbial inactivation in foods, extending their shelf life while maintaining their nutritional and sensory qualities [142]. Microbial cells are destroyed by creating irreversible pores in the membrane, inducing permeabilization and structural changes in the membrane, which may enhance the mass transfer process through the membrane and result in the release of intracellular contents and the deactivation of microorganisms [143].

Several studies have evaluated the possibility of using PEF processing to improve the shelf life stability of IFs and BFs. In a study conducted by Pina-Pérez et al. [101], the effect of PEF processing at different treatment times (60 to 3895 µs) and field strengths (10 to 40 kV/cm) on the inactivation of *C. sakazakii* suspended in buffered peptone water (BPW) and PIFM was investigated. They observed a 2.7 log_10_ (CFU/mL) reduction in *C. sakazakii* inoculated in BPW after PEF treatment for 360 µs (2.5 µs pulse width) at 40 kV/cm. In PIFM, PEF processing under the same conditions resulted in a 1.2 log (CFU/mL) reduction of *C. sakazakii*. Higher bacteria inactivation was observed in both substrates with greater field strength and treatment time. PEF processing was suggested as a promising technique to improve the safety of reconstituted IFs before storage in the refrigerator in hospitals.

Another study examined the potential cell damage of PEF processing (15 and 35 kV/cm with minimum, medium, and maximum input energy) to *C. sakazakii* inoculated in different commercial infant formula milk products [101]. The growth of survivors and the potential presence, recovery, or death of sublethally damaged cells were assessed during a 24-h refrigerated storage period at 8 °C. The utilization of PEF treatment resulted in significant damage to a large percentage (80–90%) of *C. sakazakii* cells, making them susceptible to subsequent refrigerated storage in infant formula milk. The most substantial reduction (2.30 log cycles) in *C. sakazakii* was observed with a 15 kV/cm 3000 μs PEF treatment followed by storage at 8 °C for 24 h. The reduction in cell count was mainly due to the PEF treatment, along with the formation and subsequent death of damaged cells during the refrigerated storage period.

Pina-Pérez et al. [102] investigated the synergistic effect of polyphenol-rich cocoa powder (CocoanOX 12%: CCX) and PEF processing on the deactivation of *C. sakazakii* in IFs. The study evaluated different concentrations of cocoa powder (1%, 2.5%, and 5% *w*/*v*) and the timing of cocoa powder addition (0, 2, and 4 h) before and after PEF treatment (at 15, 25, and 35 kV/cm). The goal was to assess the impact of these variables on the deactivation of *C. sakazakii* and the subsequent changes in the treated cells during refrigerated conditions (8 °C, 12 h). The results indicated that the combined application of PEF and CCX, along with the timing of CCX addition, significantly influenced the deactivation of *C. sakazakii* and the subsequent changes in the treated cells during refrigerated storage. The highest level of deactivation (4.41 log_10_ cycles) was achieved when CCX was added 4 h after PEF treatment (15 kV/cm for 3000 µs), followed by storage at 8 °C for 12 h.

A qualitative study was carried out by Nielsen et al. [144] to assess consumers’ attitudes towards novel processing technologies like PEF and HPP, and their impact on BFs. The findings indicated that participants generally held favorable views of these technologies. They perceived PEF- and HPP-treated products as more natural, nutritionally rich, tastier, and environmentally friendly. However, concerns regarding insufficient information about the technologies, skepticism, health considerations, and higher product costs were identified as the main drawbacks associated with PEF- and HPP-treated products.

## 6. Regulatory Landscape of Non-Thermal Processing Technologies

The adoption of non-thermal processing technologies like US, PEF, HPP, and RF in food production is remarkably influenced by regional regulatory frameworks. These regulations assess the approval, implementation, and labeling of foods processed with these emerging technologies. In the United States, the FDA plays a central role in regulating non-thermal technologies. For instance, HPP has gained notable traction and regulatory acceptance for utilization in various food categories, including BFs and IFs. The FDA requires detailed evidence of safety and efficacy, predominantly concerning microbial inactivation and nutritional quality preservation. Any process must comply with the FDA Food Safety Modernization Act (FSMA), ensuring that foods produced by non-thermal techniques meet stringent safety standards [145,146]. In Europe, the EFSA oversees the regulation of non-thermal technologies. This EU-funded agency evaluates new processing methods under the Novel Foods Regulation, requiring comprehensive safety assessments. Some of these technologies, like HPP and PEF, are generally recognized, provided that safety and quality are maintained without introducing harmful byproducts. However, EFSA demands robust scientific evidence to demonstrate the safety of any food processed using these methods, mainly when intended for vulnerable populations like infants [145]. In regions such as Asia and South America, the regulatory landscape is more variable. Countries like Japan and South Korea have well-defined regulations that increasingly accommodate non-thermal technologies, driven by consumer demand for minimally processed foods. In contrast, in many developing countries, the regulatory frameworks are either still evolving or lack specific guidelines for non-thermal methods. As a result, the commercialization of these technologies in certain regions can be hindered due to some reasons such as including high initial investment requirements, restricted access to reliable electricity and clean water, variations in food regulations across different countries, as well as unclear regulatory pathways and inconsistent enforcement [147]. These differences in the available regulations to apply non-thermal technologies in processing food products show the importance of establishing internationally harmonized standards to promote the global acceptance and implementation of non-thermal processing technologies in the food industry.

## 7. Conclusions and Future Trends

Several studies have demonstrated the efficacy of non-thermal processing technologies, such as high hydrostatic pressure, radio frequency, ultrasound, and pulsed electric field, in deactivating key pathogens in infant and baby products, while also reducing the formation of harmful compounds like furans. Some of these methods, particularly when combined with mild heat treatments, have shown more significant potential than when used individually. However, it is important to note that certain non-thermal technologies, such as pulsed light, supercritical fluid, microfluidization, and plasma technology, remain relatively underexplored in the context of IFs and BFs. Additionally, most research in this area has been conducted at the laboratory or pilot plant level, where conditions may differ significantly from industrial-scale production, including variations in temperature, time, pressure, and the mass or volume of food products. Validation procedures for legal acceptance and advancements in packaging technologies are also crucial considerations. Furthermore, establishing standardized definitions and labeling conventions for infant foods processed with these technologies is essential to ensure clarity and foster consumer trust. Overall, continued research and development in this area are needed to optimize the application of non-thermal processing methods in the production of safe, nutritious, and high-quality IFs and BFs.

## Figures and Tables

**Table 1:** A selection of different commercial infant formula and baby foods currently available on the market.

Type of Infant/Baby Foods	Infants’ Foods Available	Features	Stage	Ref.
First infant formula milk (IFM) powder/liquid	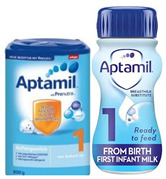	- Suitable from birth - Based on whey protein for easier digestion - Whey/casein ratio of 60:40 - Carbohydrate: lactose is the main source - Fat: polyunsaturated fatty acids and phospholipid contributes to cognitive functions, memory and concentration, cellular membrane composition, and general well-being	Stage 1	[22]
Follow-on formula (stage 2)	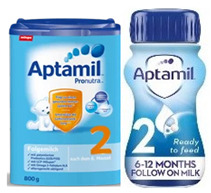	- Only suitable for babies over 6 months old as a complementary to weaning - Casein-based, they are more like cow’s milk than breast milk - In comparison to unmodified cow’s milk, these products should contain less protein, a higher fat content similar to human milk fat, and more carbohydrates. - Higher iron, calcium, zinc, and vitamin A and C levels than standard formulas, although these are less bioavailable - A major difference between IF and FOF (follow-on formula) is the higher minimum and maximum iron content of IF - Contain omega 3 and 6 and iodine for growth as well as prebiotics (GOS/FOS) and probiotics	Stage 2	[23,24]
Growing up milk GUM (toddler milk)/follow-up formula (FUF)	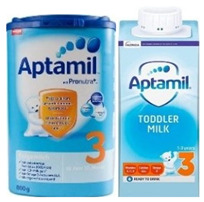	- Suitable for children between 1 and 3 years - An alternative to whole cow’s milk that compensates for nutritional deficiencies during a child’s transition to family nutrition - A GUM made from cow’s milk should preserve calcium, B_2_, and A while having a lower protein and fat content and energy value - It contains ARA and DH since the central nervous system continues to depose these compounds in high concentrations until the second year of life	Stage 3	[25]
Hungry baby formulas	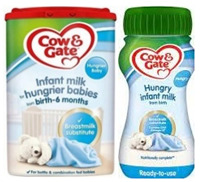	- Suitable for “hungrier babies” - Infant milk for hungrier babies is a nutritionally complete breastmilk substitute with a different balance of milk protein - Based on casein content for slow digestion and being less hungry soon after feeding - Casein based, whey/casein ratio 20:80 - Contains DHA (omega 3) as required by the legislation for all infant formula	Stage 1, 2	[26]
Preterm formula	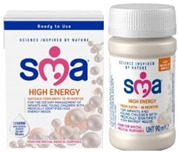	- Suitable for babies with malnutrition, growth failure - Designed for low-weight premature infants, mainly enriched with elements to continue the proper development of the nervous system - Nutrient-dense formula to support catch-up growth (100% whey protein)	Stage 1	[22]
Lactose-free formula	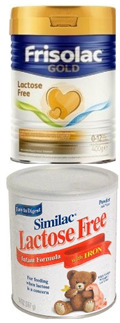	- Suitable for lactose-intolerant babies: Deficiency of the lactase enzyme prevents lactose digestion - It is not suitable for infants with galactosemia - Reduces symptoms including nausea, abdominal pain, bloating, fussiness, and gas - It contains all the necessary vitamins, minerals, trace elements, fatty acids, and DHA - Lactose-free milk is produced by adding lactase to regular cow’s milk - Lactose enzymes convert lactose into glucose and galactose, two simple sugars that make lactose-free milk sweeter than regular milk	Stage 1, 2, 3	[27]
Comfort formula	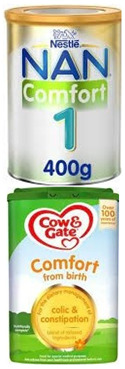	- Suitable for babies with delicate tummies - Infant digestive discomfort is the result of gastrointestinal tract immaturity, with minor digestion issues (e.g., colic, and constipation). - Partially hydrolyzed protein, easy to digest - Whey-based (100%) - With 2′-FL-HMO and nucleotides to support the baby’s immune system - A combination of supplements can relieve colic in infants (e.g., oligosaccharides, probiotics, and digestive enzymes)	Stage 1, 2, 3	[28,29]
Extensively hydrolyzed formula	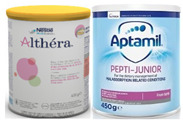	- Suitable for babies with cow’s milk protein allergy (CMPA) - Extensively hydrolyzed - Peptide-based (containing hydrolysates of casein or whey)	Stage 1, 2, 3	[30]
Hypoallergenic or elemental formula	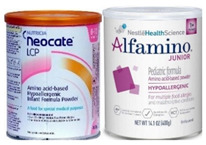	- Suitable for babies with cow’s milk protein allergy (CMPA) - Fully hydrolyzed protein - Amino acid-based formula - No residual protein	Stage 1, 2, 3	[30]
SOYA alternative milk formula	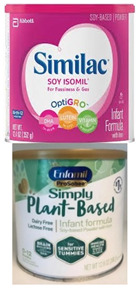	- Suitable for babies with CMPA - Alternative for extensively hydrolyzed-based formulas - It is not advised for babies under 6 months due to the development of significant osteopenia in preterm infants fed soy formula - It is not generally recommended until 1 year - There are some theoretical concerns over soya milk as it contains phytoestrogens, which adversely affect human development, reproduction, or endocrine function	Stage 3	[31]
MCT formula	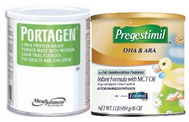	- Suitable for babies with fat malabsorption problems - With a high intake of medium chain triglycerides (MCT, 55% of fat) - The addition of functional ingredients, such as omega 3 and 6 fatty acids (docosahexaenoic acid (DHA) and arachidonic acid (ARA))	Stage 1, 2	[32]
Anti-reflux or pre-thickened formulas	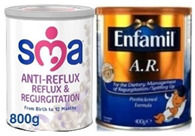	- Suitable for babies with reflux and GERD (gastroesophageal reflux disease) - The main gelling or thickening agent is carobel (e.g., carob bean gum) - Feed thickener reduces GERD by increasing the viscosity or “stickiness” of the liquid content, enabling the feed to be retained in the stomach - Formulas contain starch, vegetable fats, and 100% whey, with partially hydrolyzed protein to reduce reflux symptoms (e.g., regurgitation/spitting up)	Stage 1, 2	[33]
Probiotic infant formula	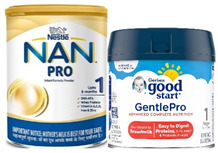	- Newborns’ gut microbiota is dominated by *Bifidobacterium* and *Lactobacillus* - Probiotics improve digestion, increase natural resistance to infectious intestinal diseases, enhance immunity, reduce cancer risks, improve nutrient synthesis and bioavailability, prevent allergies, protect the mucosa from pathogen colonization, and balance the intestinal microbiota - PIFM supplemented with probiotics at levels of 10^2^ to 10^5^ CFU/g modulates their immune systems in a similar manner to breast milk	Stage 1, 2, 3	[34,35]
PKU formula	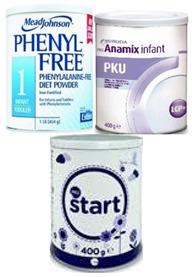	- Suitable for infants with phenylketonuria (PKU) - PKU children must follow a low-protein diet and avoid aspartame - PKU formula should be used along with breast milk or infant formula to provide the infant with phenylalanine, fluid, and general nutrition needs - Free of essential amino acid phenylalanine - Containing other essential and non-essential amino acids, fats, carbohydrates, vitamins, minerals, ARA, and DHA. - Several formulas contain glycomacropeptides - The levels of B vitamins for cofactor production are higher than in routine infant formulas	Stage 1, 2, 3	[36]
Puree meal (animal foods)	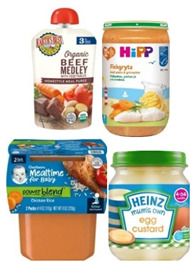	- After the first stages of life, toddlers’ feeding includes cheese and other dairy products, egg yolks, and meat as the main sources of fatty acids - High-quality protein sources should be offered to infants from 6 months, such as fish, yogurt, pureed meat, and eggs	Stage 2, 3	[37]
Organic infant formula	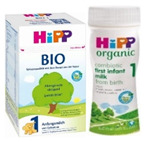	- Removing certain ingredients (e.g., palm oil, refined sugars, carrageenan, DHA, and ARA extracted using hexane), synthetic preservatives, and artificially synthesized or chemically extracted nutrients (e.g., lycopene, lutein, nucleotides, taurine, L-methionine, and L-carnitine)	Stage 1, 2, 3	[38]
Complementary foods: cereal/ porridge	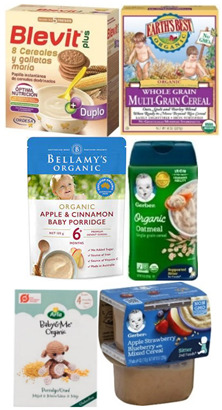	- The range of products includes rehydration-ready powders, RTS (thermally sterilized) purees formulated with cereals, dates, honey, bananas, and biscuits, as well as organic fruit and cereals. - By enzymatic dextrinization, these products promote better nutrient absorption by adapting cereal composition to infant digestion - The first stage of semisolids begins with digestible cereals, primarily rice - A puree made from whole grains (wheat, rice, barley, oats) is a good source of carbohydrates, fiber, iron, folate, and B vitamins - A porridge contains 5–8 cereals (wheat, millet, sorghum, rice, oats, barley, and rye) to deliver high nutritional value, high in fiber, calcium, and iron, and high in dextrinization (88% to 90%) - High biological value proteins, including a mixture of quinoa, cereals, and fruits rich in vitamins A, K, D, E, C, B_1_, B_2_, B_6_, biotin, folic acid, and B_12_	Stage 2, 3	[39]
Complementary foods: fruit/vegetable puree	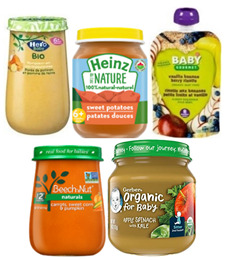	- By the age of 6 months, children are introduced to additional carbohydrate sources via pulses, fruits and vegetables, and grains - The most popular vegetable puree includes (e.g., spinach, peas, carrot, tomato sweet potato, corn, pumpkin, broccoli puree) - Fruit purees with or whit out particles (e.g., apple, banana, strawberry, mango, passion fruit, blueberry, peach) - The WHO bans added sugars in these products for children under 36 months of age	Stage 2,3	[40]
Baby water	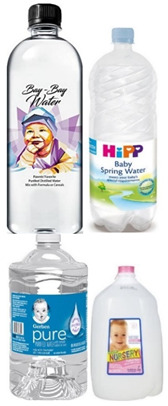	- Purified water: filtered water, with impurities removed - Suitable water for drinking when babies get older - Distilled water: made by boiling purified water and collecting steam - Distilled water is the cleanest and purest water available - This water is completely free of everything, even natural minerals such as calcium and magnesium are also removed during the process - Mineral and spring water: water derived from a spring or underground source contains naturally dissolved minerals (at least 250 ppm), including potassium, calcium, and iron	Stage 1, 2, 3	[41]

**Table 2:** The application of non-thermal processing in infant/baby food products ^1^.

Technology	Treatment Conditions	Food System or Product Substrate	Novel Processing Effects/Applications	Ref.
HHP	200, 300, and 400 MPa; 25, 35, and 45 °C; 5, 10, and 15 min	BFs Fruit puree	- Around 6 log_10_ CFU/mL inactivation of total yeasts/molds and mesophilic aerophiles at 400 MPa, 45 °C for 15 min without furan formation	[90]
HHP	200, 300, and 400 MPa; 25, 35, and 45 °C; 5, 10, and 15 min	BFs Vegetable puree	- Complete inactivation of total yeasts/molds and mesophilic aerophiles at 400 MPa, 45 °C for 15 min - No furan was detected in all treated samples	[91]
HHP	300 MPa; 20 °C; 5, 10, 15, and 20 min	IFs Based on soy protein isolate (SPI)	- Decrease in foaming stability with pressure and time increase - Decrease in emulsification stability with pressure increase - Increase in springiness, hardness, and adhesive force with time and pressure increases - Treated SPI had higher swallowing properties and digestibility	[92]
HHP	400 MPa, 20 °C, 20 min	BFs Fruit jam	- Complete inactivation of *Listeria monocytogenes* - Preserving sensory characteristics due to low temperature	[93]
HPP-TC	600 MPa, 5 min + TC (0.1%) Storage: 23 and 7 °C	RPIFM	- Significant antimicrobial activity by deactivating vegetative cells and spores of *Bacillus cereus* at both temperatures during the storage - Reduction of 2 and 3 log_10_ units of the pathogen population by HP and HP + TC	[94]
HPP CH + TC	600 MPa, 5 min + TC (0.1%) Storage at 7, 23, and 45 °C	IFs	- Without HHP: reduction of 2.1, 1.1, and 3.7 logs CFU/mL at 7, 23, and 45 °C - With HHP: at least 5.5 logs CFU/mL and total elimination of *C. sakazakii* during storage	[94]
HPTP	600 MPa, 90 °C, 5 min	BFs Pear puree	- Complete peroxidase deactivation - Maximum 60% deactivations of polyphenol oxidase	[95]
HPTS	600 MPa, 90–121 °C, 0.45–28 min	BFs Vegetable puree	- *G. stearothermophilus* was more pressure-sensitive than *B. amyloliquefaciens* at 90 and 105 °C - Furan decrease (81% to 96%)	[96]
HPTS	600 MPa, 100–115 °C, 0.45–28 min	BFs Vegetable puree	- Inactivation of 12 log_10_ CFU/mL *B. amyloliquefaciens* - Decrease in furan (41% to 98%)	[97]
PES-OH	PES: 600 MPa; 105, 110, and 121 °C for 5–10 min OH: 12 kHz; 115, 121, 125, and 130 °C for 3, 7, 14, and 21 min	BFs Carrot based puree	- Both technologies achieved lower C values - Retention of color, bioactive compounds (carotenoids), and texture was improved - Food processing contaminants (furan and its derivatives) were reduced	[98]
PATS	135 MPa, 140 °C	BFs Apple puree	- Lower decimal reduction time for *B. subtilis* - Better ascorbic acid retention	[99]
PEF	10 to 40 kV/cm/60–3895 µs/25 °C	PIFM	- 1.2 log reduction in *C. sakazakii* at 40 kV/cm, for 360 µs - The highest inactivation was obtained by increasing field strength and treatment time	[100]
PEF	15 and 35 kV/cm Storage: 8 °C, 12 h	PIFM	- Maximum inactivation of *C. sakazakii* was 2.30 log_10_ CFU/mL - The optimum condition was 15 kV/cm and 3000 μs and refrigeration for 24 h at 8 °C	[101]
PEF + CocoanOX 12%, CCX	15, 25, and 35 kV/cm	Cacao milk	- Maximum inactivation of *C. sakazakii* was 4.41 log_10_ cycles - The optimum condition for high inactivation was at 15 kV/cm for 3000 ms, and the addition of CCX 4h after PEF and refrigeration (8 °C) for up to 12 h	[102]
RF-TTP	6 kW, 27.12 MHz, 65 °C, after 21 h	PIFM	- Decrease of 5 log_10_ CFU/mL *C. sakazakii* - Significant effect on moisture content, water activity, TBArs, and peroxide value - The come-up time at 65 °C was very fast (around 10 min)	[103]
RFDH	3 kW; 27.12 MHz; at 75, 80, 85, or 90 °C; for 0 to 80 min	PIFM Nonfat dry milk (NDM)	- The D-values of *C. sakazakii* and *Salmonella* at the same temperatures were similar (*p* > 0.05), except for *C. sakazakii* at 80 °C - *C. sakazakii* and *Salmonella* spp. were destroyed as predicted	[104]
RF-HA	6 kW; 27.12 MHz; 65 and 70 °C	PIFM	- *C. sakazakii* thermal resistance was decreased with a rising water activity (0.2–0.4) at 25 °C and 55–70 °C	[105]
RF-HW	6 kW; 27.12 MHz; 65 and 70 °C	PIFM	- The development of the Maillard reaction was weak, with less color deterioration and more stable protein structures	[106]
RF-HW	60, 65, 70 and 75 °C	PIFM	- D-values for samples with a water activity of 0.3 at 60, 65, 70, and 75 °C were 77.9, 50.3, 29.9, and 15.6 min; Z-value was 21.5 °C - One log inactivation of *C. sakazakii* was found during 70 °C come up	[107]
RF-MWH	10 MHz–3 GHz; 0.36–0.54 g/cm^3^; 20–80 °C	PIFM	- PIFM can be heated uniformly between 65 °C and 3.5% by controlling the RF temperature and moisture content - Dielectric loss is positively affected by lactose and whey protein, while negatively impacted by fat	[108]
RF	27.1 MHz; 6 kW; 90 °C; 5 and 10 min	PIFM	- Better flowability and decreased compressibility - More protein dityrosine, surface-free fat, and porosity in the powder matrix	[109]
RF	10 to 3000 MHz, 20–80 °C	PIFM	- Dielectric loss decreases as fat content increases but increases as whey protein and lactose increase - Dipole loss of whey protein, fat, and lactose increased with increasing frequency - Whey protein content increases the heating rate and decreases lipid oxidation	[110]
RF	70 °C for 0, 23.3, 46.6, 69.9, 93.2, and 116.5 min	PIFM	- Water activity was decreased, while lactose crystallinity and surface free fat content remained unchanged - Particle size increased due to surface-free fat bridges after 23.3 min - Fat coverage reduced due to fat solidification as time increased from 23.3 to 116.5 min	[111]
US	20 kHz; 24.4, 30.5, 42.7, 54.9, and 61 μm; 25, 35, and 50 °C; 0, 4, 8, 12, 16, and 20 min	IFs	- Both types of *C. sakazakii* were reduced to 6.86 and 7.04 log_10_ units at 61 μm, 50 °C, within 2.5 min, and reduced to 4 log_10_ units at 25 °C within 10 min	[112]
US	750 W; 20 kHz; 25, 45, and 65 °C; 10 min	Pear puree	- Higher inactivation of yeast/mold and total plate count and enzymes at a lower temperature (65 °C, 10 min) - Higher retention of phenolic compounds and ascorbic acid	[22]
US	200, 400, or 600 W; 20 kHz; 4 °C; 15, 30, and 45 min	Meat puree	- The optimum condition was 600 W for 15 min - The treated samples had a better texture, higher water content, and lower viscosity and hardness	[113]

^1^ BFs: baby foods; CocoanOX 12% (CCX): cocoa powder; HW: combined RF and hot water treatment; HHP: high hydrostatic pressure; HPP: high-pressure processing; HPTP: high-pressure thermal processing; IFs: infant formulas; MWH: microwave heating; OH: ohmic heating; PATS: pressure-assisted thermal sterilization; PES: pressure-enhanced sterilization; PIFM: powdered infant formula milk; RF: radio frequency; RPIFM: reconstituted powdered infant formula milk; RF-HA: combined RF and hot air treatment; RF + MWH: combined RF and microwave heating; RF-TTP: combined RF with traditional thermal processing; PEF: pulsed electric field.

## Data Availability

This review article does not include any original data. We also acknowledge that there are cartoon faces depicted in two images. These cartoon faces are fictional and do not represent any real individuals. Therefore, no consent for publication was required for these images.

## Abbreviations

BFs	Baby foods
CAGR	Compound annual growth rate
FAO	Food and agriculture organization
FPCs	Food processing contaminants
FOF	Follow-on formula
FPC	Food-processing contaminants
FUF	Follow-up formula
HACCP	Hazard analysis critical control point
HHP	High hydrostatic pressure
HMF	5-hydroxymethyl-2-furfural
HPP	High-pressure processing
HPTS	High-pressure thermal sterilization
IB	Infant botulism
IFs	Infant formulas
MWH	Microwave heating
OH	Ohmic heating
PATS	Pressure assisted thermal sterilization
PEF	Pulsed electric field
PFUF	Powdered follow-up formulas
PIF	Powdered infant formula
PIFM	Powdered infant formula milk
RDA	Recommended dietary Allowance
RF	Radio frequency
RPIFM	Reconstituted powdered infant formula milk
UHT	Direct ultrahigh temperature
US	Ultrasound
